# Pre‐ Versus Post‐Breeding Population Projection Models? A Simple Fix to a Common Parametrization Error

**DOI:** 10.1002/ece3.73932

**Published:** 2026-07-01

**Authors:** Thomas L. P. Martin, Jean‐Michel Gaillard, Christophe Bonenfant

**Affiliations:** ^1^ UMR CNRS 5558—Laboratoire de Biométrie et de Biologie Évolutive, UCB Lyon 1—Bât. Grégor Mendel 43 Bd du 11 Novembre 1918 69622 Villeurbanne Cedex Villeurbanne France

## Abstract

Population Projection Matrices (PPM) are discrete mathematical formulations of population growth that are key tools in demographic population studies, enabling the prediction of growth for populations structured with age, size, or other life stages. However, several studies have identified errors stemming from misconceptions related to survival and reproduction parameters, as well as confusion between post‐breeding and pre‐breeding in the case of Leslie matrices. The present work aims to propose a simple and robust method called Matrices Alternated Products (MAP) for reliably constructing PPM directly from life tables and empirical data. The MAP approach provides a systematic framework for defining both survival and reproductive parameters and producing pre‐breeding and post‐breeding matrices. The method we propose allows reducing errors and improving the accuracy of demographic population models, and the code we provide will allow population ecologists and wildlife managers to apply this method to any case study in practice.

## Graphical abstract

Population Projection Models (PPMs) are essential tools in demographic studies for predicting the growth of structured populations. This work introduces a simple, robust method for constructing pre‐ and post‐breeding projection matrices called MAP for Matrices Alternated Products directly from empirical data, addressing common errors related to survival and reproduction parameters. By providing a systematic framework and an accompanying R package, our approach improves the accuracy of PPMs and facilitates their application in ecological and wildlife management contexts.

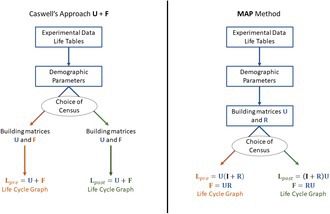

## 1 Introduction

Population Projection Matrices (PPM), also known as matrix models, are discrete mathematical formulations of population growth (Leslie 1945, 1948). PPM have been playing a pivotal role in the study of the dynamics and demography of structured populations (Caswell 2001). Originally introduced by P.H. Leslie in 1945 (Leslie 1945, 1948), PPMs gained in popularity among ecologists in the late 80s, providing insights into the age‐ or stage‐specific growth and stability of plant and animal populations (Tuljapurkar 1990; Lande et al. 2003; Caswell 2001). Serving as a cornerstone for demographic analyses in research fields ranging from ecology (Flipse and Veling 1984; Nordheim et al. 1989) to conservation biology (Lebreton and Clobert 1991; Slooten and Lad 1991), and evolutionary biology (Van Groenendael et al. 1988; Demetrius et al. 2007), PPMs now underpin many advanced population modeling tools such as integral projection (Easterling et al. 2000).

If the construction of a PPM and its associated life‐cycle graph is rather straightforward (e.g., Caswell 2001), the parameterization of its entries from empirically estimated demographic rates appears to be an error‐prone step for many users. In PPMs, what values should be used for offspring production and transition between stages at each time step does not directly match with the observed demographic rates. A review by Kendall et al. (2019) searched and quantified the rate of errors and recurring issues in building PPMs in previously published studies of population dynamics. As much as 30% of the reviewed case studies used a wrongly parameterized PPM (Kendall et al. 2019). Such errors in the parameterization of PPMs are not trivial and can markedly affect conclusions of the studies (Mollet and Cailliet 2003). Among others, errors in PPM parameterization could lead to misinterpretation of population dynamics, to erroneous population projections over time, and to biases in asymptotic growth rate estimates (Kendall et al. 2019). Although the magnitude and sign of such errors on the estimation of the asymptotic or stochastic population growth rates remain largely unknown, some conservation or wildlife management actions have likely been under‐evaluated or unnecessary implemented (see examples from the Appendix of Kendall et al. (2019)).

The main challenge when building a PPM arises from the need to combine two events in a specific order and unit them into PPM parameters (Kendall et al. 2019). The first event is reproduction which, in PPMs, is treated as an instantaneous event often referred to as birth pulse (Leslie 1945). This number can be called fecundity, the capacity for an individual to produce offspring. Obviously, this birth pulse assumption does not accurately reflect the reproductive biology of all species; in fact, because many organisms, such as humans, reproduce continuously throughout the year in a pattern known as “birth flow”. Nevertheless, populations exhibiting birth pulse dynamics are by far the most naturally suited to the discrete‐time structure of PPMs. For species characterized by continuous birth flows, continuous demographic approaches (such as continuous‐time structured models) are generally more appropriate (Caswell 2001). The second event is survival, which is a continuous process that unfolds over the interval between two time points at which the population census takes place. Reproduction is instantaneous while survival is measured over a pre‐defined time interval, often corresponding to 1 year in vertebrate demography. This difference in the scale of measurement makes it difficult to reconcile reproduction with survival within a single modeling framework.

**FIGURE 1:**
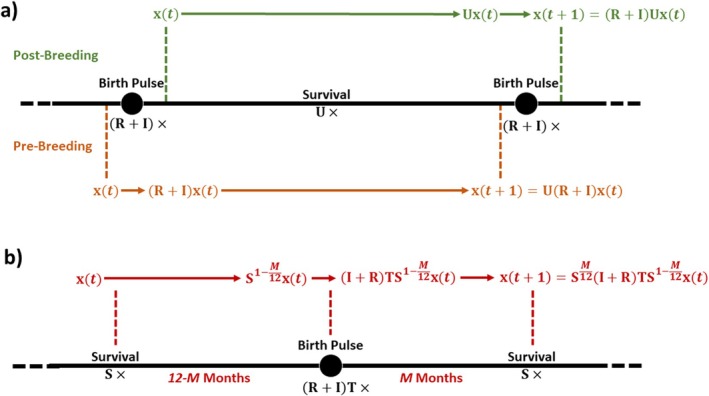
(a) Schematic illustration of the difference between counting individuals immediately before (*bottom*: Pre‐breeding census) or after (*top*: Post‐breeding census) the birth pulse. **x**(t) is the population vector at time t. Birth pulse is described mathematically by left‐multiplying **x** by matrix (***R*** + **I**), while survival over 1 year is represented by left‐multiplication by matrix **U**. (b) Schematic illustration of the difference when counting individuals between two birth pulses (M months after birth pulse, intermediate census). **x**(t) is the population vector at time t. Birth pulse is described mathematically by left‐multiplying **x** by matrix (***R*** + **I**) *T*, while survival over M months is represented by left‐multiplication by matrix $S^{\frac{M}{12}}$.

When evaluating the reproduction rate between two successive censuses, the number of offspring produced by the parent during the birth pulse also includes the survival rate of the offspring (from its birth to the next census) or of the parent (from the census to parturition) over the year. The solution to this issue lies in choosing the appropriate timing for population censusing. Depending on when the animals are censused relative to the reproduction, two parameterizations of a PPM are possible: pre‐breeding and post‐breeding (see Figure 1a):

- **Post‐breeding census PPM:** The population is censused immediately after reproduction. In this case, offspring are newborns when censused, so their survival is assumed to be one when estimating reproduction rates. However, the survival of the parents between the previous and current census must be included because only females that survived this time interval can give birth to newborns. Consequently, the reproduction rate is the product of the fecundity and the survival probability of the parent age‐class.
- **Pre‐breeding census PPM:** In this case the population is censused just before reproduction. Here, parent survival should not be included in the recruitment rate—individuals ready to reproduce are censused right before giving birth and are, by definition, still alive. However, the offspring survival from birth to the next census (i.e., survival to 1 year of age when censuses are 1 year apart) has to be included in the recruitment part.

When correctly written, post‐ and pre‐breeding PPMs yield the same dynamics and estimate of population growth rate (Caswell 2001), however, the sensitivity and elasticity can be different. For age‐structured populations, a well known consequence of choosing a pre‐breeding over a post‐breeding formulation is the loss of one dimension because newborns are never observed when census takes place right before reproduction. This choice of a PPM may also imply a different biological interpretation of analyses, such as sensitivity analyses (see Mollet and Cailliet 2003). This distinction is, however, a common source of mistakes, often arising from confusion between pre‐ and post‐breeding parameterizations, or from cases where survival is not accounted for in reproduction rates at all (Kendall et al. 2019). The choice of post‐breeding census can lead to higher error rates in the parameterization of PPMs (the overall error rate of 30% in the cases studies by Kendall et al. (2019) increases to 60% when restricted to the post‐breeding cases).

According to Caswell (2001), one way to build a PPM is to sum two matrices **U** and **F**, where **U** contains survival rates and **F** contains reproductive rates associated with the appropriate survival probabilities of parents or offspring, according to the timing of census relative to reproduction. Survival rates are therefore already multiplied by the reproduction rate, meaning that the choice between pre‐ and post‐breeding is known and implicit to compute PPM entries. This does not alleviate the problem of choice and correct parametrization.

In this work, we present an explicit and systematic method called Matrix Alternated Products (MAP) to generate **U** and **F** matrices for both the pre‐ and post‐breeding PPMs using two intuitive matrices that includes observed survival and reproduction rates only. The utility of this framework is twofold for applied ecologists. Theoretically, it establishes a rigorous mathematical framework to formally demonstrate fundamental demographic properties. Notably, this formalism yields an analytical proof for the strict invariance of the asymptotic population growth rate (λ) between pre‐ and post‐breeding formulations. It also mechanistically explains the main structural differences, such as the disappearance of the youngest age‐class in age‐structured pre‐breeding models. Practically, it provides a transparent and alternative protocol that reduces the risk of parameterization errors.

This modeling framework is readily extendable to more complex population structures and is applicable to alternative models with stage‐structure (Lefkovitch 1965), social structure (Rézouki et al. 2016), body mass classes (Gamelon et al. 2012) or other structuring factors. In these more complex cases, reproduction still occurs within the matrix, but it is no longer confined to the first row and can occur in multiple classes leading to no loss of dimension for the pre‐breeding formulation. We also show how the MAP can generate a correct PPM when censuses occur at any time relative to the reproduction event. We hope this approach, applicable to a wide array of biological systems and life cycles, will contribute to mitigate these frequently reported errors in building PPMs.

## 2 From Demographic Rates to PPMs

In practice, the experimental data for transition rates between classes, survival rates in age‐structured models, are expressed over a time interval. If the time interval between two censuses is 1 year, the dimension of transition rates is year^−1^ as well as the one of fecundity (Lindstedt et al. 1986). The point of the MAP method is to store those data in two different matrices, **U** and **R**, from which one generates a pre‐ or post‐breeding PPM. Unlike **U** and **F** (Caswell 2001), **U** and **R** are not added to build a PPM. We show below how a matrix product of **U** and **R**, however, returns the same PPMs as the addition of **U** and **F**. The risk of confusion between the two PPM parameterizations is reduced because the values stored in **U** and **R** do not depend on the choice of a pre‐ or post‐breeding PPM.

For a system with n classes, the matrix **U** $={\left(u_{\mathrm{ij}}\right)}_{1\leq i,j\leq n}$ describes the transition between classes where $u_{\mathrm{ij}}$ is the transition rate from the class j to the class i. Note in the case of a simple age‐structured PPM, the transition always goes from i to i+1 over 1 year so $s_{i+1,i}$ is generally noted $s_{i}$. The matrix **R** $={\left(r_{\mathrm{ij}}\right)}_{1\leq i,j\leq n}$ describes the fecundity rate of each class where $r_{\mathrm{ij}}$ indicates the number of individuals of class i produced by an individual from class j at a given time. Again, in a age‐structured PPM, new individuals always appear in the first class 1 so the fecundity rates $r_{1,i}$ are generally given as $r_{i}$. Note that by convention matrix index starts at 1 so for an age‐structured PPM, class 1 describes individuals aged between 0 and 1 years old.

We can use **U** and **R** matrices to compute both post‐ and pre‐breeding PPMs. The population with n classes at time t is described by a n− dimensional vector **x** $\left(t\right)$ where $x_{i}\left(t\right)$ is the number of individuals of class i at time t. Mathematically, the reproduction event can be described as a left multiplication of the vector **x** $\left(t\right)$ by the matrix ( **I** + ***R*** ), the identity matrix **I** appears because the reproduction event only creates new individuals so those already present stay alive after the event. Similarly, transitions are represented by a left multiplication of **x** $\left(t\right)$ by the matrix **U**.

In a pre‐breeding PPM reproduction occurs first followed by the class transition over a time interval. Using this mathematical formalism described above, the associated projection matrix $L_{\mathrm{pre}}$ is described as:

(1) $$L_{\mathrm{pre}}≔U\left(I+R\right).$$

Conversely, in the post‐breeding census, the class transition occurs first over one time interval followed by the reproduction. Here the associated projection matrix $L_{\text{post}}$ then becomes:

(2) $$L_{\text{post}}≔\left(I+R\right)U$$

The key difference between our **U** and **R** matrix‐based approach and the **U** and **F** matrice‐based approach proposed by Caswell (2001) is the implicit census choice (pre or post‐breeding PPMs) of the latter while **U** and **R** offer the flexibility of yielding both formulations from simple matrices of demographic rates. In all cases the **U** matrices are the same and therefore expanding the formulas (1) and (2) reveals the correspondence between our method and the **U** + **F** decomposition, which depends on the selected timing of census with regard to reproduction. In the pre‐breeding census **U** = **U** and **F** = **UR**. In the post‐breeding census, **U** = **U** and **F** = **RU**. This is illustrated in Caswell (2001)'s example 4.1, where a corrected matrix is proposed: although the reproductive terms differ because of the choice in the census timing, the survival components remain unchanged in the new matrix (see p.60 Caswell 2001).

This approach can be generalized beyond traditional pre‐ and post‐breeding censuses and adapted to situations where the population is monitored outside of the breeding period (Cooch et al. 2003) (see Figure 1b). Pre‐ and post‐breeding PPMs both consider individuals at the beginning of their age‐class. When intermediate situations occur, individuals are of intermediate age at the time of census. For instance, when a census is performed three months before the timing of births, then individuals observed in age‐class i are in fact i years old +9 months. In order to model such cases, we suggest a slightly different decomposition of the survival matrix **U** into two matrices **S** and **T**. **S** is a diagonal matrix including survival rates of each age‐class. **T** includes the transition between ages with a simple sub‐diagonal identity matrix. With this decomposition, we can handle intermediate census timings (see Section 3.2).

A general formulation is that if the census is taken M months after births, individuals of class i are i years‐old and M months old and the PPM is given by $L=S^{\frac{M}{12}}$ (**I** + ***R***) **T** $S^{1-\frac{M}{12}}$. The post‐breeding case is simply the limiting case where M=0. However, the pre‐breeding PPM is not the limiting case when M=12, because when M=12 censusing individuals at the very end of their annual interval, classified in age‐class i would in fact be almost i+1 years old, which is a strongly unrealistic scenario from a biological viewpoint. Another interest of splitting the survival process into two matrices is the possibility to handle seasonal variation in survival. For instance, if survival rates differ before and after the censuses, different survival matrices **S** could be used instead of partitioning the annual matrix uniformly, and to assume constant survival over a year. It should be noted that in the case of intermediate censuses, the additive **U** + **F** decomposition no longer yields to the same **U** matrix, in fact **L** = $S^{\frac{M}{12}}$ (**I** + ***R***) **T** $S^{1-\frac{M}{12}}$ = $S^{\frac{M}{12}}$ **T** $S^{1-\frac{M}{12}}$ + $S^{\frac{M}{12}}$ **RT** $S^{1-\frac{M}{12}}$, the first term of the sum is not equal to **U**. Consequently, drawing a direct correspondence with Caswell's framework is no longer feasible.

## 3 Application of MAP to Simple Cases

### 3.1 Computations of Pre‐ and Post‐Breeding PPMs From a Life Table

A widely used tool by demographers and biologists in the case of age‐class models are the life tables (Deevey Jr 1947). A life table gives for each age a two values, $p\left(a\right)$ that is the probability to reach the age a and $m\left(a\right)$ that is the fecundity rate of age a, the number of offspring produced by an individual of age a. An example of a simple life table is given in Table 1. In an age‐structured model, the only possible transitions are those from one age a to the next a+1, corresponding to aging over one single time step. In this case, the transition rate is given by the survival probability of the age a. This implies that the non‐zero entries of **U** have the form $u_{a+2,a+1}=s_{a}=\frac{p\left(a+1\right)}{p\left(a\right)}$ (if a+1> maximum age of the table, we set $p\left(a+1\right)=0$). The index shift arises from the fact that Age 0 is represented by the row 1 in the matrix. As for the matrix **R**, reproduction in such age‐specific model is restricted to individuals producing offspring of age 0, hence non‐zero entries of **R** are of the form $r_{1,a+1}=m\left(a\right)$. Again, the index shift is due to Age 0 being represented by the row 1 of the matrix. The matrices **U** and **R** corresponding to the life Table 1 are:

(3) $$U=\left(\begin{array}{cccc} 0 & 0 & 0 & 0\\ 0.5 & 0 & 0 & 0\\ 0 & 0.6 & 0 & 0\\ 0 & 0 & 0.7 & 0 \end{array}\right),R=\left(\begin{array}{cccc} 0 & 1.5 & 2.5 & 2.5\\ 0 & 0 & 0 & 0\\ 0 & 0 & 0 & 0\\ 0 & 0 & 0 & 0 \end{array}\right)$$

**TABLE 1:** Theoretical example of a life table describing age‐specific demographic parameters. The first column indicates age (in years), the second gives the probability $p\left(a\right)$ of reaching that age, the third shows the average number of female offspring produced per female $m\left(a\right)$ at that age, and the fourth gives the annual survival probability $s\left(a\right)$ at that age.

Age a	$p\left(a\right)$	$m\left(a\right)$	$s\left(a\right)$
0	1	0	0.5
1	0.5	1.5	0.6
2	0.3	2.5	0.7
3	0.21	2.5	0

The projection matrices for the pre‐breeding and post‐breeding census are computed as the products of **U** and **R** as described in Section 2:

(4) $$L_{\mathrm{pre}}=U\left(I+R\right)=\left(\begin{array}{cccc} 0 & 0 & 0 & 0\\ 0.5 & 0.75 & 1.25 & 1.25\\ 0 & 0.6 & 0 & 0\\ 0 & 0 & 0.7 & 0 \end{array}\right)$$

(5) $$L_{\text{post}}=\left(I+R\right)U=\left(\begin{array}{cccc} 0.75 & 1.5 & 1.75 & 0\\ 0.5 & 0 & 0 & 0\\ 0 & 0.6 & 0 & 0\\ 0 & 0 & 0.7 & 0 \end{array}\right)$$

For a pre‐breeding PPM of an age‐structured population, the matrix usually has one dimension less compared to the post‐breeding one (Caswell 2001; Kendall et al. 2019). The reason is that when individuals are censused right before birth, the youngest are born 1 year earlier and are already 1 year old. Biologically, it is not possible to observe individuals in the first age‐class under these conditions. In our example, the pre‐breeding matrix $L_{\mathrm{pre}}$ has the same dimension as $L_{\text{post}}$. Because the MAP method should handle any cases, so we do not automatically drop one dimension in the pre‐breeding PPM. Although this situation is common, exceptions holds, for instance when juvenile class lasts for more than 1 year. Nonetheless, in this example, we can remove one dimension from the matrix to be consistent with the biological interpretation with no effects on the dynamics.

(6) $$L_{\mathrm{pre}}=\left(\begin{array}{ccc} 0.75 & 1.25 & 1.25\\ 0.6 & 0 & 0\\ 0 & 0.7 & 0 \end{array}\right)$$

Similarly, in the case of the post‐breeding census, the dimension of the matrix can often be reduced by removing the last age‐class without altering the model's asymptotic dynamics (Leslie 1945; Lefkovitch 1965). From a biological perspective, although individuals in this terminal age‐class are physically present and observable at the time of the census t, they will not survive until the next breeding season t+1. Since they can no longer contribute to the population's future reproduction or survival, they have no influence on the subsequent dynamics. Mathematically, it appears in the last column of $L_{\text{post}}$ full of zeros, allowing for its removal with the last row without loss of information. However, this simplification only holds for strict age‐classified models; it is invalid if the last category is a group aggregating all individuals above a certain age as these individuals may survive and continue to reproduce, thereby maintaining a functional role in the population's trajectory.

(7) $$L_{\text{post}}=\left(\begin{array}{ccc} 0.75 & 1.5 & 1.75\\ 0.5 & 0 & 0\\ 0 & 0.6 & 0 \end{array}\right)$$

**FIGURE 2:**
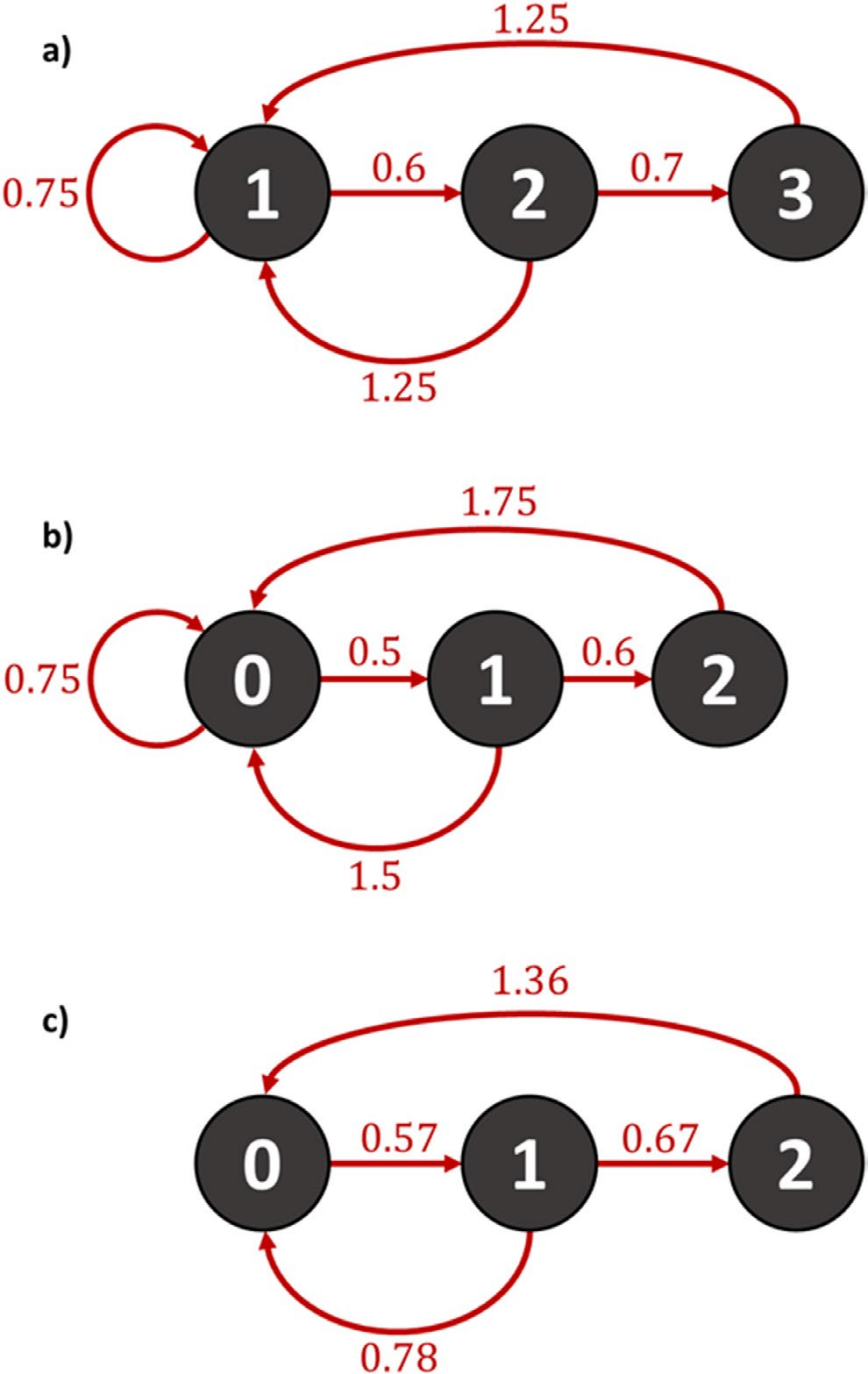
(a) The life‐cycle graph derived from the pre‐breeding PPM for the age structured model described by the life table (Table 1). (b) The life‐cycle graph derived from the post‐breeding PPM for the age structured model described by the life table (Table 1). (c) The life‐cycle graph derived from the intermediate PPM in which the census takes place 3 months before birth pulse for the age structured model described by the life table (Table 1).

**FIGURE 3:**
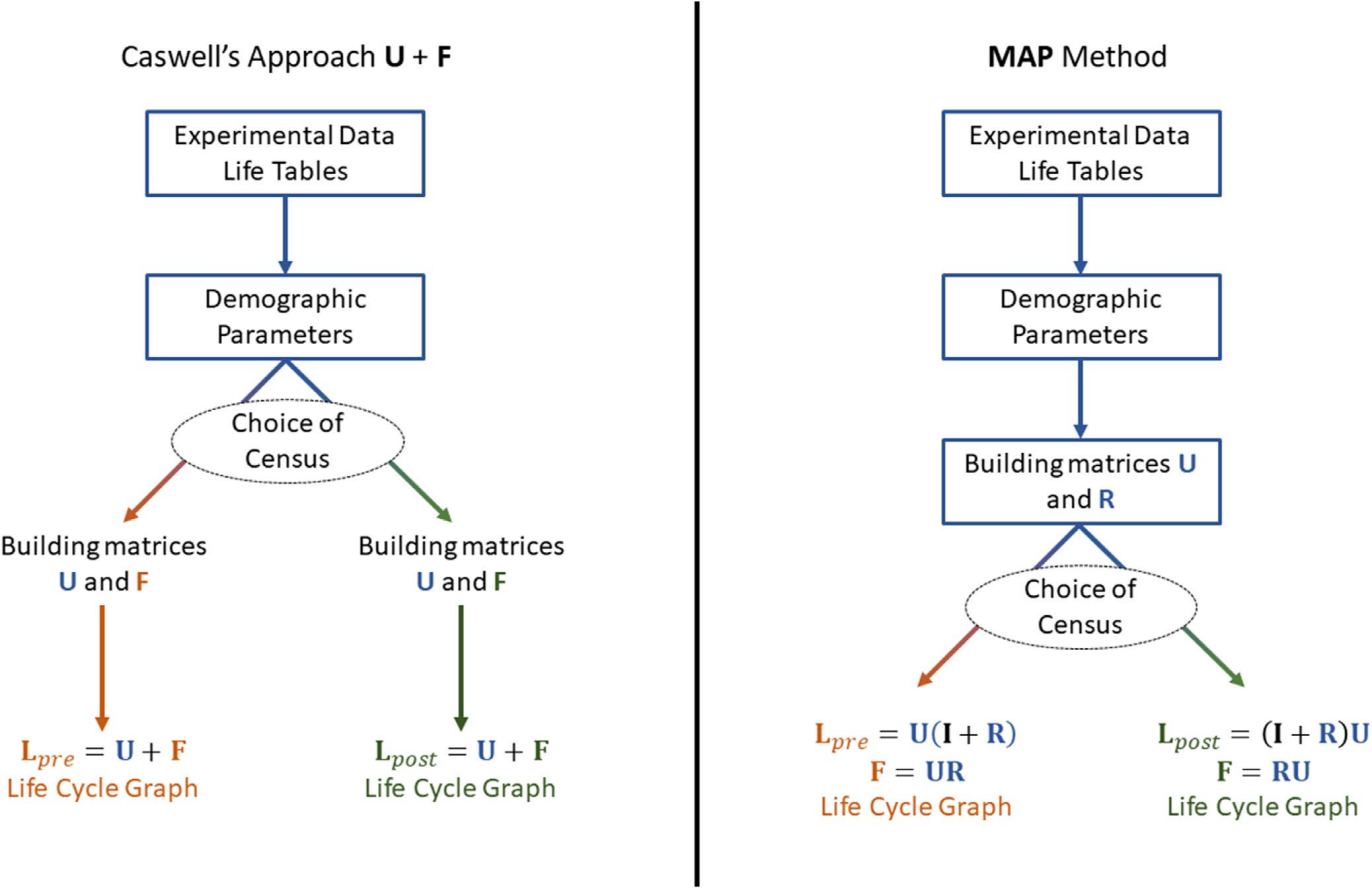
*Comparison between the classical* **U** + **F** *approach and the MAP method*: In the **U** + **F** approach (*left*), demographic parameters extracted from experimental data or life tables are used to construct **U** and **F** matrices according to either a pre‐ or post‐breeding census scheme. This step, often prone to error, determines the rest of the analysis: The resulting matrices are used to build the PPM ant the life‐cycle graphs. However, the **F** matrix is specific to the chosen census type and cannot be reused for the other. In contrast, the MAP method (*right*) uses demographic parameters to directly construct the **U** and **R** matrices, independently of any census choice. Pre‐ and post‐breeding PPMs are then derived by simple matrix product, from which life‐cycle graphs are generated. The key advantage of this method is that **U** and **R** matrices are built linearly from the demographic parameters, inserted directly into the matrices without calculations, and can be used for any type of census schemes as well as for the PPM.

The associated life‐cycle graphs for the pre‐ and post‐breeding PPMs are displayed in Figures 2a & 2b, respectively. Note that in this case, the characteristic polynomial of $L_{\text{post}}$ is the Euler‐Lotka equation associated with the life Table 1 so its dominant eigenvalue is the asymptotic growth rate, this is one way to derive the Euler‐Lotka equation (Keyfitz and Caswell 2005). All the steps and the comparison between the standard approach **U** + **F** and the approach proposed here are summarized in the Figure 3. A comprehensive and more complex age‐structured example using empirical data from roe deer ( *Capreolus capreolus* ) (Gaillard et al. 1993, 2013) is provided in the Appendix S4. In this example, demographic paramters for survival and reproduction were estimated using the method described in Nilsen et al. (2009).

### 3.2 Asynchronous Census and Reproduction

As an example, let us take the life Table 1 and place the census 3 months before births. In this case, the needed matrices are:

$$S=\left(\begin{array}{cccc} 0.5 & 0 & 0 & 0\\ 0 & 0.6 & 0 & 0\\ 0 & 0 & 0.7 & 0\\ 0 & 0 & 0 & 0 \end{array}\right),T=\left(\begin{array}{cccc} 0 & 0 & 0 & 0\\ 1 & 0 & 0 & 0\\ 0 & 1 & 0 & 0\\ 0 & 0 & 1 & 0 \end{array}\right),R=\left(\begin{array}{cccc} 0 & 1.5 & 2.5 & 2.5\\ 0 & 0 & 0 & 0\\ 0 & 0 & 0 & 0\\ 0 & 0 & 0 & 0 \end{array}\right).$$

Biologically, **S** includes the survival probability of each class over 1 year, **T** includes the class transition (from one age‐class i to the next i+1), and **R** is the same as described in Section 2, the reproduction matrix, which includes reproduction rate as a birth pulse. To track population dynamics correctly, we must first account for survival during the initial 3 months, then apply the transition to the next age‐class and reproduction, and finally survival over the remaining 9 months. The corresponding PPM is computed as follows:

$$L_{\mathrm{int}}=S^{\frac{3}{4}}\left(I+R\right)TS^{\frac{1}{4}},$$

where for all 0≤α≤1:

$$S^{\alpha }=\left(\begin{array}{cccc} 0.5^{\alpha } & 0 & 0 & 0\\ 0 & 0.6^{\alpha } & 0 & 0\\ 0 & 0 & 0.7^{\alpha } & 0\\ 0 & 0 & 0 & 0^{\alpha } \end{array}\right),$$

which is mathematically correct because the matrix **S** is diagonal. Using this formula the desired PPM for this situation where censuses occur 3 months before reproduction is as follows:

$$L_{\mathrm{int}}=\left(\begin{array}{cccc} 0.75 & 1.31 & 1.36 & 0\\ 0.57 & 0 & 0 & 0\\ 0 & 0.67 & 0 & 0\\ 0 & 0 & 0 & 0 \end{array}\right).$$

This matrix returns the same asymptotic growth rate as the pre and post‐breeding PPMs (λ=1.49) and the life graph cycle shown in the Figure 2c. It is worth noting that, in this intermediate representation, the last row and column of the matrix are zeros and can be omitted. Mathematically, this reflects the fact that the final age‐class has no further influence on the model and can therefore be excluded. This representation of the population dynamics is also biologically consistent: if i denotes the upper age limit of the model, individuals aged i years and 9 months cannot be observed.

### 3.3 Case of Life Cycles With Self‐Looping Stages

While life tables constitute a widely used and practical tool among biologists and ecologists, they remain limited in scope, as they apply only to age‐structured models. The MAP approach proposed here overcomes this limitation and can also be applied to models that are not strictly age‐structured. As an example, let us consider a system composed of three Classes 1, 2, and 3 which includes self‐loop transition (transition from one class to itself). Individuals of Class 1 stays in Class 1 with transition rate $u_{11}$ and moves to Class 2 with a rate $u_{21}$, individuals of Class 2 stays in Class 2 with transition rate $u_{22}$ and moves to Class 3 with a rate $u_{32}$ and individuals of Class 3 stays in Class 3 with a transition rate $u_{33}$. Only individuals in Classes 2 and 3 reproduce in Class 1, with respective reproduction rates $r_{2}$ and $r_{3}$. Such a model can be used to describe situations where the juvenile stage lasts for more than a year, as for example for the frog (Vonesh and De la Cruz 2002). For such a system the pre‐ and post‐breeding PPMs are as follows:

(8) $$L_{\mathrm{pre}}=\left(\begin{array}{ccc} u_{11} & u_{11}r_{2} & u_{11}r_{3}\\ u_{21} & u_{21}r_{2}+u_{22} & u_{21}r_{3}\\ 0 & u_{32} & u_{33} \end{array}\right),L_{\text{post}}=\left(\begin{array}{ccc} u_{11}+u_{21}r_{2} & u_{22}r_{2}+u_{32}r_{3} & u_{33}r_{3}\\ u_{21} & u_{22} & 0\\ 0 & u_{32} & u_{33} \end{array}\right)$$

All details regarding the calculations of these two matrices, as well as a validation of their accuracy by comparing the obtained results with an explicit computation of the population dynamics of the same system over one time step, are provided in Appendix S2.

## 4 Application to a Complex Life‐Cycle

**FIGURE 4:**
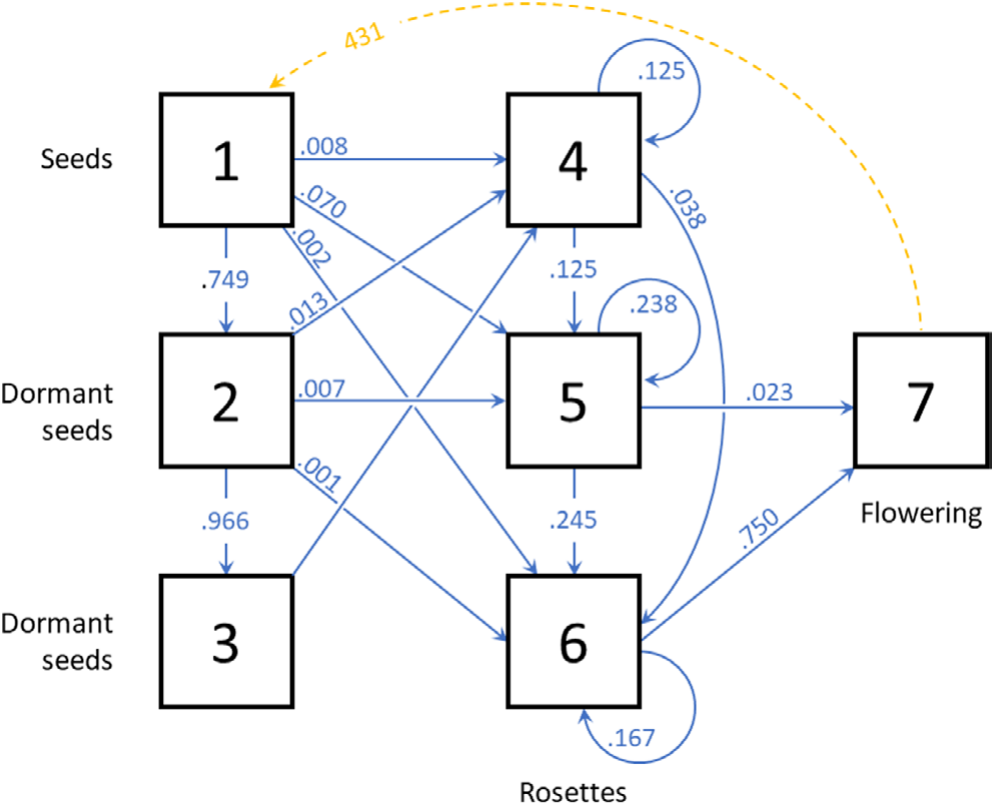
Graphical representation for *Dispacus sylvestris* based on, field A, stage classification in Table 3 of Werner and Caswell (1977). Stages: $N_{1}=$ seeds, $N_{2}=$ first‐year dormant seeds, $N_{3}=$ second‐year dormant seeds, $N_{4}=$ small rosettes, $N_{5}=$ medium rosettes, $N_{6}=$ large rosettes, and $N_{7}=$ flowering plants. Arrows with coefficient equal to 0 were removed. Dashed yellow lines describe reproduction rates (as birth pulse). Full blue lines describe transition rates.

A slightly more complex example is described by Caswell (2001), who revisited the plant‐based models of *Dispacus sylvestris* originally coined by Werner and Caswell (1977) (table 3, Field A, Stage‐classification). The graph of Figure 4 does not correspond to a life‐cycle graph but simply is a visual representation of the possible transitions among states of the model. In this case, the temporal mismatch between reproduction and survival/transition processes is particularly striking, as highlighted by Caswell (2001): Stage 1, which corresponds to seeds, does not span a full year. As a result, combining fecundity (i.e., seed production) with survival and transition rates within a single matrix leads to a two‐year delay from flowering to the production of dormant seeds (Stage 2 or 3), which is an unrealistic outcome (Caswell 2001). To resolve this issue, the authors adopted a pre‐breeding census approach, assessing the population state just before the production of new seeds and allowing one full year for seed transitions to occur. In doing so, the seed class (Stage 1) was excluded from the matrix and also from their life‐cycle graph. This approach is valid and coherent; however, our method generalizes this framework. The matrix transformation used by Caswell (2001) involves multiplying reproductive outputs by the relevant transition probabilities and placing the resulting values in the last column of the new matrix. This is exactly what we obtain in our pre‐breeding formulation using the product of matrices **R** and **U**. Importantly, the MAP method also enables the construction of the associated post‐breeding census matrix, in which the seed class (Stage 1) is retained. Both approaches yield identical population growth rates, highlighting that the choice between them reflects a modeling or biological perspective rather than any error associated with the use of a post‐breeding census for this specific life cycle.

In this system, the only reproductive event links the flowering (Stage 7) producing seeds (Stage 1), therefore the only non‐zero entry of the reproduction matrix **R** is the reproduction rate $r_{1,7}=431.0$. All the other terms of the graph are part of the transition matrix **U** with the rule given in the Section 2. The matrices **R** and **U** are as follow:

(9) $$R=\left(\begin{array}{ccccccc} 0 & 0 & 0 & 0 & 0 & 0 & 431.0\\ 0 & 0 & 0 & 0 & 0 & 0 & 0\\ 0 & 0 & 0 & 0 & 0 & 0 & 0\\ 0 & 0 & 0 & 0 & 0 & 0 & 0\\ 0 & 0 & 0 & 0 & 0 & 0 & 0\\ 0 & 0 & 0 & 0 & 0 & 0 & 0\\ 0 & 0 & 0 & 0 & 0 & 0 & 0 \end{array}\right)$$

(10) $$U=\left(\begin{array}{ccccccc} 0 & 0 & 0 & 0 & 0 & 0 & 0\\ 0.749 & 0 & 0 & 0 & 0 & 0 & 0\\ 0 & 0.966 & 0 & 0 & 0 & 0 & 0\\ 0.008 & 0.013 & 0.010 & 0.125 & 0 & 0 & 0\\ 0.070 & 0.007 & 0 & 0.125 & 0.238 & 0 & 0\\ 0.002 & 0.001 & 0 & 0.038 & 0.245 & 0.167 & 0\\ 0 & 0 & 0 & 0 & 0.023 & 0.750 & 0 \end{array}\right)$$

The PPMs for both pre‐ and post‐breeding censuses are computed from those matrices, the PPM in the pre‐breeding case is $L_{\mathrm{pre}}$ = **U** × (**I** + ***R***) and the PPM in the post‐breeding case is $L_{\text{post}}$ = (**I** + ***R***) × **U**. Applied to numerical values displayed in Caswell's work the matrices for this system are:

(11) $$L_{\mathrm{pre}}=U\left(I+R\right)=\left(\begin{array}{ccccccc} 0 & 0 & 0 & 0 & 0 & 0 & 0\\ 0.748 & 0 & 0 & 0 & 0 & 0 & 322.39\\ 0 & 0.966 & 0 & 0 & 0 & 0 & 0\\ 0.008 & 0.013 & 0.010 & 0.125 & 0 & 0 & 3.448\\ 0.07 & 0.007 & 0 & 0.125 & 0.238 & 0 & 30.17\\ 0.002 & 0.008 & 0 & 0.038 & 0.245 & 0.167 & 0.862\\ 0 & 0 & 0 & 0 & 0.023 & 0.750 & 0 \end{array}\right)$$

(12) $$L_{\text{post}}=\left(I+R\right)U=\left(\begin{array}{ccccccc} 0 & 0 & 0 & 0 & 9.913 & 323.25 & 0\\ 0.748 & 0 & 0 & 0 & 0 & 0 & 0\\ 0 & 0.966 & 0 & 0 & 0 & 0 & 0\\ 0.008 & 0.013 & 0.010 & 0.125 & 0 & 0 & 0\\ 0.07 & 0.007 & 0 & 0.125 & 0.238 & 0 & 0\\ 0.002 & 0.008 & 0 & 0.038 & 0.245 & 0.167 & 0\\ 0 & 0 & 0 & 0 & 0.023 & 0.750 & 0 \end{array}\right)$$

In the equations (11) and (12) the terms in red correspond to reproduction events, they depend on the choice of census and therefore differ between the pre and post‐breeding matrices. It is worth noting that our pre‐breeding matrix $L_{\mathrm{pre}}$ differs slightly from Caswell (2001)'s corrected matrix $A_{2}$. While it is, of course, possible to omit the first row and column, as previously discussed, some differences remain. These arise from an inconsistency within the corrected matrix itself, based on Caswell's own criteria. Although the corrected matrix is intended to differ from the original matrix only in the last column, the term $a_{5,3}$ that should have been retained as 0.038 was incorrectly set to 0 (not that we used the value 0.038 of the original article by Werner and Caswell (1977) instead of the value 0.036 used by Caswell (2001)). In practice, Caswell (2001)'s corrected matrix should match our pre‐breeding matrix $L_{\mathrm{pre}}$ (without the first row and column). In the post‐breeding framework, the issue of the short lifespan of seeds was addressed by shifting seed production from the flowering stage to earlier stages (rosettes stages 5 and 6). In this setup, one accounts for the transition from rosettes to flowers, followed by seed production within a single year, thereby avoiding the unrealistic two‐year delay in dormant seed production seen in the original matrix. Importantly, both matrices $L_{\mathrm{pre}}$ and $L_{\text{post}}$ have the same dominant eigenvalue and therefore provide the same estimated asymptotic population growth rate λ=2.33. We applied the MAP method for all the examples provided in (Werner and Caswell 1977) and the corrected growth rates are shown in Table 2 and the full associated PPMs are available in Table S1.

**TABLE 2:** Population growth rates (λ) obtained from population projection matrices (PPMs) for stage model of *Dipsacus sylvestris* shown in table 4 of Werner and Caswell (1977) and the corrected one for both post‐ and pre‐breeding censuses with our method. The first column (Field) represents the different study sites of the experiment, the vegetation characteristics of these sites are detailed in the table 1 of Werner and Caswell (1977).

Field	Original λ	Corrected λ
A	1.797	2.334
B	1.989	2.478
C	1.875	2.247
D	2.071	2.546
J	0.628	0.516
K	0.275	0.275
L	1.195	1.258
M	2.605	3.793

## 5 Discussion

That PPMs can be parameterized differently according to the way the transition rates have been collected is known since at least Keyfitz and Caswell (2005) (first edition 1977) who already highlighted this issue in 1977. Still, post‐ and pre‐breeding formulations of the same PPM seem to be error‐prone for many users (Kendall et al. 2019). In the specific case of age‐class PPMs, one can find unpublished material most of the time offering case‐specific solutions, but we have been lacking a formalization and generalization of the way to do it so far. Thanks to a partition between a reproduction matrix and a survival matrix, the MAP approach should help in building pre‐ and post‐breeding PPM for any life cycle, even with complex life histories as frequently displayed by plant species for instance. However, careful attention must still be paid to the way empirical data are gathered, as field protocols and sampling timing can directly dictate how PPMs are constructed. The MAP method is specifically designed to accommodate class transition and fecundity data as they are most commonly collected and reported in traditional life tables commonly published in the ecological literature.

In the overwhelming majority of ecological studies, obtaining a complete and exhaustive census of a wild population is practically unattainable. Consequently, population dynamics are predominantly assessed through the estimation of key demographic parameters, most notably survival and reproduction rates. To capture these metrics accurately, ecologists deploy a wide spectrum of analytical and experimental tools, allowing them to reconstruct the broader demographic reality from partial field observations. Ultimately, the construction and parameterization of PPMs depend heavily on the specific data made available through all these combined methods (Alberts and Altmann 2003).

A confusion may arise between the **R** and **U** matrices defined in this paper, and previous types of decomposition of projection matrices. In open access databases of projection matrices like Comadre (e.g., Salguero‐Gómez et al. 2016, for animal species) and Compadre (e.g., Salguero‐Gómez et al. 2015, for plant species), or some population dynamics packages, package mpmsim for R, Jones (2025), reproductive and survival rates are stored and retrieved as separate matrices corresponding to **U** and **F** of Caswell (2001). To reconstruct the original projection matrix, **U** and **F** matrices must be summed, whereas the product is computed here. This distinction is crucial and has important implications for building the formulation of the PPM right. The reproductive rates should be expressed in the same temporal dimension as survival rates, meaning that annual rates apply to survival from the parent to give birth, the survival of the offspring starting at birth, or a combination thereof. In other words, the reproductive rates used in these additive approaches are not raw fecundities as estimated from biological data such as pregnancy rates or reproductive success; rather, they already incorporate a temporal structure aligned with either a pre‐breeding or a post‐breeding framework.

Additionally, class transitions that represent the probability that an individual moves from class i to class j within one time step interval, replace the simpler survival‐only transitions of age‐structured models. In the age‐structured PPM framework, this transition reduces to the survival probability, whereas in more general stage‐structured PPM, a broader range of transitions is captured leading to more complex PPM construction. In fact, conventional generalizations of PPMs typically reduce the pre‐breeding matrix by one. While this assumption holds true in many situations (especially standard age‐structured models), it cannot be universally applied to all stage‐structured models and requires additional assumptions. The MAP minimizes confusion and provides a general framework applicable to any type of class structure of population.

Finally, it is worth emphasizing that while the choice of census timing alters the mathematical structure of the PPM, it preserves the fundamental population‐level properties. Specifically, as we saw, the asymptotic population growth rate, but also other key demographic metrics derived from the underlying demographic parameters such as generation time, Keyfitz's entropy (Keyfitz and Caswell 2005), and Demetrius's entropy (Demetrius 1992), remain invariant to the choice of a pre’ or post‐breeding formulation. This paper come with a companion R package called ppmconstruction (a comprehensive presentation and user guide for this package are provided in the Appendix S5). By providing a natural and mathematically consistent correspondence between post‐ and pre‐breeding formulations, the MAP approach effectively bridges the gap between field‐estimated vital rates and formal demographic modeling for applied demography and applied ecology.

## Author Contributions

**Thomas L. P. Martin:** conceptualization (equal), methodology (lead), software (lead), writing – original draft (lead). **Jean‐Michel Gaillard:** data curation (lead), supervision (supporting), writing – review and editing (equal). **Christophe Bonenfant:** conceptualization (equal), data curation (lead), funding acquisition (lead), supervision (lead), writing – original draft (supporting), writing – review and editing (equal).

## Funding

This work was supported by Institut écologie et environnement, SEE‐life and Agence Nationale de la Recherche, ANR SOCIALIPOP.

## Conflicts of Interest

The authors declare no conflicts of interest.

## Supporting information


**Figure S1:** ece373932‐sup‐0001‐FigureS1.tif.

**Data S1:** ece373932‐sup‐0002‐Supinfo.pdf.

## Data Availability

All data used in this article are directly drawn from sources cited in the paper for the examples with experimental data. The R package and codes used are available at https://github.com/StankTLPM/ppmpackage.git.
