# Supplementary figures and images for: Pre‐ Versus Post‐Breeding Population Projection Models? A Simple Fix to a Common Parametrization Error
Source: Ecol Evol. 2026 Jul 1;16(7):e73932. doi: 10.1002/ece3.73932 (PMC13322775; doi:10.1002/ece3.73932)

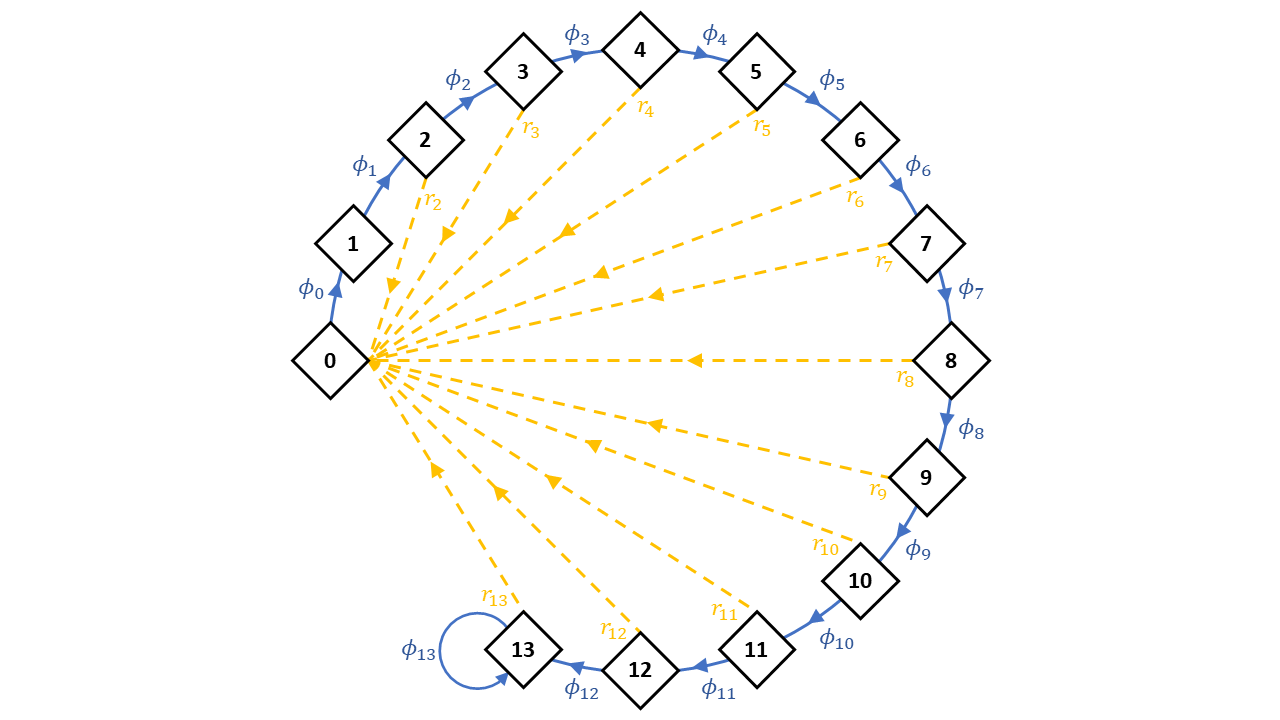

Supplement: Supplementary file 1 — Figure S1: ece373932‐sup‐0001‐FigureS1.tif. [file ECE3-16-e73932-s001.tif]
