# Supplementary material for: Pre‐ Versus Post‐Breeding Population Projection Models? A Simple Fix to a Common Parametrization Error
Source: Ecol Evol. 2026 Jul 1;16(7):e73932. doi: 10.1002/ece3.73932 (PMC13322775; doi:10.1002/ece3.73932)
Supplement: Supplementary file 2 — Data S1: ece373932‐sup‐0002‐Supinfo.pdf. [file ECE3-16-e73932-s002.pdf]

S1 Dynamics comparison of reduced and full PMMs

Mathematically, if the first row of the matrix is zero, this implies that the first coefficient of the population vector (representing the first age-class) will become zero at every time step, as can be verified:

$$\begin{pmatrix} 0 & 0 & 0 & 0 \\ 0.5 & 0.75 & 1.25 & 1.25 \\ 0 & 0.6 & 0 & 0 \\ 0 & 0 & 0.7 & 0 \end{pmatrix} \times \begin{pmatrix} x_1 \\ x_2 \\ x_3 \\ x_4 \end{pmatrix} = \begin{pmatrix} 0 \cdot x_1 + 0 \cdot x_2 + 0 \cdot x_3 + 0 \cdot x_4 \\ 0.5 \cdot x_1 + 0.75 \cdot x_2 + 1.25 \cdot x_3 + 1.25 \cdot x_4 \\ 0 \cdot x_1 + 0.6 \cdot x_2 + 0 \cdot x_3 + 0 \cdot x_4 \\ 0 \cdot x_1 + 0 \cdot x_2 + 0.7 \cdot x_3 + 0 \cdot x_4 \end{pmatrix} = \begin{pmatrix} 0 \\ 0.5 \cdot x_1 + 0.75 \cdot x_2 + 1.25 \cdot x_3 + 1.25 \cdot x_4 \\ 0.6 \cdot x_2 \\ 0.7 \cdot x_3 \end{pmatrix}$$

Removing the first age-class therefore does not change the system, since it will always remain zero, which makes sense because biologically it is impossible to observe those individuals. Consequently, the first row and column of the matrix can be eliminated, even if there are non-zero entries in the column, because they only serve as multiplicative coefficients of the first component of the population vector which itself is null:

$$\begin{pmatrix} 0 & 0 & 0 & 0 \\ 0.5 & 0.75 & 1.25 & 1.25 \\ 0 & 0.6 & 0 & 0 \\ 0 & 0 & 0.7 & 0 \end{pmatrix} \times \begin{pmatrix} 0 \\ x_2 \\ x_3 \\ x_4 \end{pmatrix} = \begin{pmatrix} 0 \cdot 0 + 0 \cdot x_2 + 0 \cdot x_3 + 0 \cdot x_4 \\ 0.5 \cdot 0 + 0.75 \cdot x_2 + 1.25 \cdot x_3 + 1.25 \cdot x_4 \\ 0 \cdot 0 + 0.6 \cdot x_2 + 0 \cdot x_3 + 0 \cdot x_4 \\ 0 \cdot 0 + 0 \cdot x_2 + 0.7 \cdot x_3 + 0 \cdot x_4 \end{pmatrix}$$

$$= \begin{pmatrix} 0 \\ 0.75 \cdot x_2 + 1.25 \cdot x_3 + 1.25 \cdot x_4 \\ 0.6 \cdot x_2 \\ 0.7 \cdot x_3 \end{pmatrix}$$

This category thus naturally disappears from the calculations, and biologically, this reflects the fact that individuals younger than one year cannot be observed in the pre-breeding census. Using the matrices $\mathbf{L}_{pre}$ and $\mathbf{L}_{post}$, we can draw life cycle graphs for both censuses as shown in Figure 2a for the pre-breeding census and in Figure 2b for the post-breeding one.

S2 Computation and Validation of PPMs for a Three-Class Model Including Self-Loop Transitions

In this part, we demonstrate how the presented method can be applied to a simple yet non-trivial case involving 3 different classes with self-loop transition. To illustrate the relevance of our approach, we first manually compute the evolution of the system we describe, and then apply, step by step, the method proposed in this article. Both approaches lead to exactly the same results. We consider a system composed of three classes 1, 2, and 3, individuals of class 1 stay in class

1 with transition rate t_{11} and moves to class 2 with a rate t_{21}, individuals of class 2 stay in class 2 with transition rate t_{22} and moves to class 3 with a rate t_{32} and individuals of class 3 stay in class 3 with a transition rate t_{33}. Only individuals in classes 2 and 3 reproduce in class 1, with respective reproduction rates r_2 and r_3. The population at time t is described by a 3-dimensional vector $\mathbf{x}(t) = (x_1(t), x_2(t), x_3(t))^T$.

In the pre-breeding census case, the coefficients of the population vector $\mathbf{x}(t+1)$ according to the ones of $\mathbf{x}(t)$ are:

- $x_1(t+1)$**:** individuals that were in class 1 ($x_1(t)$) and stay in this class (t_{11}), individuals born from parents of class 2 ($x_2(t)r_2$) or from class 3 ($x_3(t)r_3$) that stay in the class 1 (t_{11}):

$$x_1(t+1) = t_{11}x_1(t) + t_{11}(x_2(t)r_2 + x_3(t)r_3).$$

- $x_2(t+1)$**:** individuals from class 1 ($x_1(t)$) moving to class 2 (t_{21}), individuals that were in class 2 ($x_2(t)$) and stay in this class (t_{22}), individuals born from parents of class 2 ($x_2(t)r_2$) or from class 3 ($x_3(t)r_3$) that move in the class 2 (t_{21}):

$$x_2(t+1) = t_{21}x_1(t) + t_{22}x_2(t) + t_{21}(x_2(t)r_2 + x_3(t)r_3).$$

- $x_3(t+1)$**:** individuals from class 2 ($x_2(t)$) moving to class 2 (t_{32}) and individuals from class 3 ($x_3(t)$) staying in this class (t_{33}):

$$x_3(t+1) = t_{32}x_2(t) + t_{33}x_3(t).$$

Combining all those equations, the pre-breeding census model for this system is given by :

$$\begin{pmatrix} x_1(t) \\ x_2(t) \\ x_3(t) \end{pmatrix} = \begin{pmatrix} t_{11}x_1(t) + t_{11}(x_2(t)r_2 + x_3(t)r_3) \\ t_{21}x_1(t) + t_{22}x_2(t) + t_{21}(x_2(t)r_2 + x_3(t)r_3) \\ t_{32}x_2(t) + t_{33}x_3(t) \end{pmatrix}. \tag{13}$$

In the post-breeding census case, the coefficients of the population vector $\mathbf{x}(t+1)$ according to the ones of $\mathbf{x}(t)$ are:

- $x_1(t+1)$**:** individuals that were in class 1 ($x_1(t)$) and stay in this class (t_{11}), individuals of class 1 ($x_1(t)$) moving to class 2 (t_{21}) and giving birth in this class (r_2), individuals of class 2 ($x_2(t)$) staying in this class (t_{22}) and giving birth (r_2), individuals of class 2 ($x_2(t)$) moving to class 3 (t_{32}) and giving birth in this class (r_3) and individuals of class 3 ($x_3(t)$) staying in this class (t_{33}) and giving birth (r_3):

$$x_1(t+1) = t_{11}x_1(t) + t_{21}x_1(t)r_2 + t_{22}x_2(t)r_2 + t_{32}x_2(t)r_3 + t_{33}x_3(t)r_3.$$

- $x_2(t+1)$**:** individuals from class 1 ($x_1(t)$) moving to class 2 (t_{21}), individuals that were in class 2 ($x_2(t)$) and stay in this class (t_{22}):

$$x_2(t+1) = t_{21}x_1(t) + t_{22}x_2(t).$$

- $x_3(t+1)$**:** individuals from class 2 ($x_2(t)$) moving to class 2 (t_{32}) and individuals from class 3 ($x_3(t)$) staying in this class (t_{33}):

$$x_3(t+1) = t_{32}x_2(t) + t_{33}x_3(t).$$

Combining all those equations, the post-breeding census model for this system is given by :

$$\begin{pmatrix} x_1(t) \\ x_2(t) \\ x_3(t) \end{pmatrix} = \begin{pmatrix} t_{11}x_1(t) + t_{21}x_1(t)r_2 + t_{22}x_2(t)r_2 + t_{32}x_2(t)r_3 + t_{33}x_3(t)r_3 \\ t_{21}x_1(t) + t_{22}x_2(t) \\ t_{32}x_2(t) + t_{33}x_3(t) \end{pmatrix}. \tag{14}$$

Using our method, the matrix $\mathbf{U}$ compiled all the transitions described by the rates $u_{11} = t_{11}$, $u_{21} = t_{21}$, $u_{22} = t_{22}$, $u_{32} = t_{32}$ and $u_{33} = t_{33}$, whereas the matrix $\mathbf{R}$ compiles the reproduction rates on the first row because newborn individuals appear in the class 1 so $r_{12} = r_2$ and $r_{13} = r_3$, therefore:

$$\mathbf{U} = \begin{pmatrix} t_{11} & 0 & 0 \\ t_{21} & t_{22} & 0 \\ 0 & t_{32} & t_{33} \end{pmatrix}, \mathbf{R} = \begin{pmatrix} 0 & r_2 & r_3 \\ 0 & 0 & 0 \\ 0 & 0 & 0 \end{pmatrix}.$$

Based on the matrices $\mathbf{U}$ and $\mathbf{R}$, the PPM for the two censuses pre- and post-breeding are computed using the formulas $\mathbf{L}_{pre} = \mathbf{U}\ (\mathbf{I} + \mathbf{R})$ and $\mathbf{L}_{post} = (\mathbf{I} + \mathbf{R})\ \mathbf{U}$:

$$\mathbf{L}_{pre} = \mathbf{U}(\mathbf{I} + \mathbf{R}) = \begin{pmatrix} t_{11} & t_{11}r_2 & t_{11}r_3 \\ t_{21} & t_{21}r_2 + t_{22} & t_{21}r_3 \\ 0 & t_{32} & t_{33} \end{pmatrix}, \tag{15}$$

$$\mathbf{L}_{post} = (\mathbf{I} + \mathbf{R})\mathbf{U} = \begin{pmatrix} t_{11} + t_{21}r_2 & t_{22}r_2 + t_{32}r_3 & t_{33}r_3 \\ t_{21} & t_{22} & 0 \\ 0 & t_{32} & t_{33} \end{pmatrix}. \tag{16}$$

The model for the pre-breeding case is described as follows:

$$\mathbf{x}(t+1) = \mathbf{L}_{pre}\mathbf{x}(t) = \begin{pmatrix} x_1(t)t_{11} + x_2(t)t_{11}r_2 + x_3(t)t_{11}r_3 \\ x_1(t)t_{21} + x_2(t)t_{21}r_2 + x_2(t)t_{22} + x_3(t)t_{21}r_3 \\ x_2(t)t_{32} + x_3(t)t_{33} \end{pmatrix}, \quad (17)$$

which is exactly what we have in Equation 13. The model for the post-breeding case is described as follows:

$$\mathbf{x}(t+1) = \mathbf{L}_{post}\mathbf{x}(t) = \begin{pmatrix} x_1(t)t_{11} + x_1(t)t_{21}r_2 + x_2(t)t_{22}r_2 + x_2(t)t_{32}r_3 + x_3(t)t_{33}r_3 \\ x_1(t)t_{21} + x_2(t)t_{22} \\ x_2(t)t_{32} + x_3(t)t_{33} \end{pmatrix}, \quad (18)$$

which is exactly what we have in Equation 14.

S3 Full PPM For the Plant Life-cycle

Table S1: Fully corrected population projection matrices for *Dipsacus sylvestris* from data in Table 4 of Werner and Caswell (1977) (stage-classification) for both pre-breeding and post-breeding census. Corrections were made using our package.

**Field**	**Pre-breeding matrix**							**Post-breeding matrix**						
A	...	...	...	...	...	...	...	...	...	...	...	9.913	323.250	...
	0.748	...	...	...	...	...	322.388	0.748	...	...	...	...	...	...
	...	0.966	...	...	...	...	...	...	0.966	...	...	...	...	...
	...	0.013	0.01	0.125	...	...	3.448	...	0.013	0.01	0.125	...	...	...
	0.070	0.007	...	0.125	0.238	...	30.170	0.070	0.007	...	0.125	0.238	...	...
	0.002	0.008	...	0.038	0.245	0.167	0.862	0.002	0.008	...	0.038	0.245	0.167	...
	...	...	...	...	0.023	0.750	...	...	...	...	...	0.023	0.750	...
B	...	...	...	...	...	...	...	...	...	...	...	...	275.280	...
	0.761	...	...	...	...	...	353.865	0.761	...	...	...	...	...	...
	...	0.965	...	...	...	...	...	...	0.965	...	...	...	...	...
	0.009	0.044	0.016	...	...	...	4.185	0.009	0.044	0.016	...	...	...	...
	0.066	0.016	0.001	0.074	0.269	...	30.690	0.066	0.016	0.001	0.074	0.269	...	...
	0.004	...	...	...	0.462	0.367	1.860	0.004	...	...	...	0.462	0.367	...
	...	...	...	...	...	0.592	...	...	...	...	...	...	0.592	...
C	...	...	...	...	...	...	...	...	...	...	...	...	942.956	...
	0.756	...	...	...	...	...	855.792	0.756	...	...	...	...	...	...
	...	0.940	...	...	...	...	...	...	0.940	...	...	...	...	...
	0.011	0.033	0.003	...	...	...	12.452	0.011	0.033	0.003	...	...	...	...
	0.014	...	...	0.338	0.268	...	15.848	0.014	...	...	0.338	0.268	...	...
	...	...	...	0.045	0.513	0.167	...	...	...	...	0.045	0.513	0.167	...
	...	...	...	...	...	0.833	...	...	...	...	...	...	0.833	...

Table S1 – continued from previous page

**Field**	**Pre-breeding matrix**								**Post-breeding matrix**						
D	...	...	...	...	...	...	...		...	...	...	...	...	737.035	...
	0.567	...	...	...	...	...	626.535		0.567	...	...	...	...	...	...
	...	0.916	...	...	...	...	...		...	0.916	...	...	...	...	...
	0.031	0.053	0.018	0.018	...	...	34.255		0.031	0.053	0.018	0.018	...	...	...
	0.035	0.018	...	0.327	0.394	...	38.675		0.035	0.018	...	0.327	0.394	...	...
	...	...	...	0.056	0.376	...	...		...	...	...	0.056	0.376	...	...
	...	...	...	...	...	0.667	...		...	...	...	...	...	0.667	...
J	...	...	...	...	...	...	...		...	...	...	...	...	119.0	...
	0.423	...	...	...	...	...	201.348		0.423	...	...	...	...	...	...
	...	0.987	...	...	...	...	...		...	0.987	...	...	...	...	...
	0.024	0.009	0.006	0.007	...	...	11.424		0.024	0.009	0.006	0.007	...	...	...
	0.044	...	...	0.050	0.158	...	20.944		0.044	...	...	0.050	0.158	...	...
	0.001	...	...	0.002	0.008	...	0.476		0.001	...	...	0.002	0.008	...	...
	...	...	...	...	...	0.25	...		...	...	...	...	...	0.25	...
K	...	...	...	...	...	...	...		...	...	...	...	...	...	...
	0.800	...	...	...	...	...	...		0.800	...	...	...	...	...	...
	...	0.959	...	...	...	...	...		...	0.959	...	...	...	...	...
	0.001	0.034	0.001	...	...	...	...		0.001	0.034	0.001	...	...	...	...
	0.007	0.006	...	0.048	0.275	...	...		0.007	0.006	...	0.048	0.275	...	...
	...	...	...	...	0.025	...	...		...	...	...	...	0.025	...	...
	...	...	...	...	...	...	...		...	...	...	...	...	...	...

Table S1 – continued from previous page

Field	Pre-breeding matrix							Post-breeding matrix						
L	...	...	...	...	...	...	...	...	...	...	...	1.006	260.051	...
	0.430	...	...	...		...	216.290	0.430	...	...	...	...	...	...
	...	0.970	...	...	...	...	...	...	0.970	...	...	...	...	...
	0.010	0.021	0.005	...	...	...	5.030	0.010	0.021	0.005	...	...	...	...
	0.036	0.003	...	0.19	0.253	...	18.108	0.036	0.003	...	0.19	0.253	...	...
	...	...	...	0.07	0.105	0.150	...	...	...	...	0.07	0.105	0.150	...
	...	...	...	...	0.001	0.517	...	...	...	...	...	0.002	0.517	...
M	...	...	...	...	...	...	...	...	...	...	...	40.005	635	...
	0.634	...	...	...	...	...	402.590	0.634	...	...	...	...	...	...
	...	0.974	...	...	...	...	...	...	0.974	...	...	...	...	...
	0.013	0.017	0.011	...	...	...	8.255	0.013	0.017	0.011	...	...	...	...
	0.109	0.004	0.002	0.077	0.212	...	69.215	0.109	0.004	0.002	0.077	0.212	...	...
	0.006	0.003	...	0.038	0.281	...	3.810	0.006	0.003	...	0.038	0.281	...	...
	...	...	...	...	0.063	1	...	...	...	...	...	0.063	1	...

S4 Supplementary Materials: Application to the Roe Deer Case

In this study, we apply our method to the simple case of a standard age-structured PPM to model the population dynamics of roe deer (*Capreolus capreolus*). The life history of this species is well-documented (Gaillard et al., 1993), making it an ideal candidate for illustrating our approach. Specifically, the PPM includes age-specific reproduction rates on the first row and age-dependent survival probabilities on the sub-diagonal. By focusing on this simple case study, we aim to demonstrate the suitability of our method. The demographic parameters for survival and reproduction were estimated using the method described

in Nilsen et al. (2009). Empirical data were collected from roe deer at Chizé between 2000 and 2010.

The model examined in this section represents the roe deer population structured in 18 age-classes from 0 to 17, focusing exclusively on females, as is commonly done in PPM. To simplify the model, we aggregated the oldest age-classes (ages 13 to 17) into a single terminal class, labeled 13 as their reproductive and survival rates were similar, making this grouping biologically and analytically justified (Gaillard et al., 2013). All other age-classes, from 0 to 12, represent single-year intervals. Individuals begin reproducing at age 2. The interactions between age-classes are summarized in the biological graph in Figure S1. (note this graph is not a life-cycle graph), with numerical values provided in the accompanying table. The reproductive rates (r_i) shown in the graph correspond to fecundities at a punctual event, independently of whether the model follows a pre- or post-breeding census (also called $m(i)$, a standard metric in demography). Those fecundities are calculated as the product of three terms, the probability of a female to give birth, the average litter size, and the average female-to-male ratio of the offspring (see Table S2). The reproduction rate in post-breeding census is the product of the fecundity and the survival probability of the mother the year before, in pre-breeding census, the reproduction rate is the product of the fecundity and the survival probability the first year.

We used the companion package to MAP method we propose here to generate both pre- and post-breeding PPM for this case study of roe deer based on the model presented in the Figure S1. The matrices with symbolic parameter expressions are given in the Table S3 for the post-breeding matrix and in the Table S4 for the pre-breeding matrix. Note again that in the case of a pre-breeding census, the first row and the first column can be removed. Numerical application was also made for this case study using values from the Table S2

Table S2: Demographic parameters for female roe deer (*Capreolus capreolus*) across 14 age-classes. s_i represents the annual survival probability for age-class i, r_i is the fecundity for age-class i ($r_i = P_g \times$ litter $\times$ sex ratio), P_g is the probability of giving birth, litter refers to the average litter size, and sex ratio denotes the proportion of female offspring. The values are used to parameterize the PPM for the population.

**age-class**	s_i	r_i	P_g	**litter**	**sex ratio**
0	0.699	0	0	0.00	0.5
1	0.774	0	0	0.00	0.5
2	0.947	0.846	0.90	1.88	0.5
3	0.947	0.897	0.98	1.83	0.5
4	0.947	0.858	0.98	1.75	0.5
5	0.947	0.874	0.93	1.88	0.5
6	0.947	0.907	0.98	1.85	0.5
7	0.947	0.887	0.98	1.81	0.5
8	0.912	0.950	1.00	1.90	0.5
9	0.912	0.839	0.97	1.73	0.5
10	0.912	0.823	0.89	1.85	0.5
11	0.912	0.858	0.97	1.77	0.5
12	0.912	0.731	0.87	1.68	0.5
13	0.634	0.496	0.73	1.36	0.5

by computing the dominant eigenvalue of the two matrices. As expected from theoretical considerations, both the pre-breeding and post-breeding census matrices yielded the same value for the asymptotic population growth rate, that reads $\lambda = 1.299$.

S5 Presentation of the R package ppmconstruction

The R package we developed includes four core functions: two for handling matrices with numeric values and two preserving literal parameter expressions. For each representation, numeric and symbolic, the package provides a function to generate both pre-breeding and post-breeding projection matrices. All functions take as input three parameters: an integer specifying the number of classes in

Table S3: Post-breeding census PPM for the roe deer (*Capreolus capreolus*) population at Chizé, showing age-specific survival (ϕ_i) and reproduction (r_i).

$$
\begin{pmatrix}
r_1\phi_0 & r_2\phi_1 & r_3\phi_2 & r_4\phi_3 & r_5\phi_4 & r_6\phi_5 & r_7\phi_6 & r_8\phi_7 & r_9\phi_8 & r_{10}\phi_9 & r_{11}\phi_{10} & r_{12}\phi_{11} & r_{13}\phi_{12} & r_{13}\phi_{13} \\
\phi_0 & 0 & 0 & 0 & 0 & 0 & 0 & 0 & 0 & 0 & 0 & 0 & 0 & 0 \\
0 & \phi_1 & 0 & 0 & 0 & 0 & 0 & 0 & 0 & 0 & 0 & 0 & 0 & 0 \\
0 & 0 & \phi_2 & 0 & 0 & 0 & 0 & 0 & 0 & 0 & 0 & 0 & 0 & 0 \\
0 & 0 & 0 & \phi_3 & 0 & 0 & 0 & 0 & 0 & 0 & 0 & 0 & 0 & 0 \\
0 & 0 & 0 & 0 & \phi_4 & 0 & 0 & 0 & 0 & 0 & 0 & 0 & 0 & 0 \\
0 & 0 & 0 & 0 & 0 & \phi_5 & 0 & 0 & 0 & 0 & 0 & 0 & 0 & 0 \\
0 & 0 & 0 & 0 & 0 & 0 & \phi_6 & 0 & 0 & 0 & 0 & 0 & 0 & 0 \\
0 & 0 & 0 & 0 & 0 & 0 & 0 & \phi_7 & 0 & 0 & 0 & 0 & 0 & 0 \\
0 & 0 & 0 & 0 & 0 & 0 & 0 & 0 & \phi_8 & 0 & 0 & 0 & 0 & 0 \\
0 & 0 & 0 & 0 & 0 & 0 & 0 & 0 & 0 & \phi_9 & 0 & 0 & 0 & 0 \\
0 & 0 & 0 & 0 & 0 & 0 & 0 & 0 & 0 & 0 & \phi_{10} & 0 & 0 & 0 \\
0 & 0 & 0 & 0 & 0 & 0 & 0 & 0 & 0 & 0 & 0 & \phi_{11} & 0 & 0 \\
0 & 0 & 0 & 0 & 0 & 0 & 0 & 0 & 0 & 0 & 0 & 0 & \phi_{12} & \phi_{13}
\end{pmatrix}
$$

Table S4: Pre-breeding PPM for the roe deer (*Capreolus capreolus*) population of Chizé, showing age-specific survival (ϕ_i) and reproduction (r_i). The first row and column were removed because the first row was full of zeros.

$$
\begin{pmatrix}
\phi_0 r_1 & \phi_0 r_2 & \phi_0 r_3 & \phi_0 r_4 & \phi_0 r_5 & \phi_0 r_6 & \phi_0 r_7 & \phi_0 r_8 & \phi_0 r_9 & \phi_0 r_{10} & \phi_0 r_{11} & \phi_0 r_{12} & \phi_0 r_{13} \\
\phi_1 & 0 & 0 & 0 & 0 & 0 & 0 & 0 & 0 & 0 & 0 & 0 & 0 \\
0 & \phi_2 & 0 & 0 & 0 & 0 & 0 & 0 & 0 & 0 & 0 & 0 & 0 \\
0 & 0 & \phi_3 & 0 & 0 & 0 & 0 & 0 & 0 & 0 & 0 & 0 & 0 \\
0 & 0 & 0 & \phi_4 & 0 & 0 & 0 & 0 & 0 & 0 & 0 & 0 & 0 \\
0 & 0 & 0 & 0 & \phi_5 & 0 & 0 & 0 & 0 & 0 & 0 & 0 & 0 \\
0 & 0 & 0 & 0 & 0 & \phi_6 & 0 & 0 & 0 & 0 & 0 & 0 & 0 \\
0 & 0 & 0 & 0 & 0 & 0 & \phi_7 & 0 & 0 & 0 & 0 & 0 & 0 \\
0 & 0 & 0 & 0 & 0 & 0 & 0 & \phi_8 & 0 & 0 & 0 & 0 & 0 \\
0 & 0 & 0 & 0 & 0 & 0 & 0 & 0 & \phi_9 & 0 & 0 & 0 & 0 \\
0 & 0 & 0 & 0 & 0 & 0 & 0 & 0 & 0 & \phi_{10} & 0 & 0 & 0 \\
0 & 0 & 0 & 0 & 0 & 0 & 0 & 0 & 0 & 0 & \phi_{11} & 0 & 0 \\
0 & 0 & 0 & 0 & 0 & 0 & 0 & 0 & 0 & 0 & 0 & \phi_{12} & \phi_{13}
\end{pmatrix}
$$

the model and two lists, one for class transitions/survival and one for the fecundity, defined as the number of offspring produced on average by a female of age x at a given time. These lists follow a common structure, consisting of triplets of the form $c(i, j, p)$, representing each directed edge in the life cycle graph, where i is the origin class, j the destination class, and p the value or name of the parameter. For example, a transition from class 1 to class 2 with survival probability 0.6 is encoded as $c(1, 2, 0.6)$, while a basal reproduction from class 3 to class 1 with rate 50 is given as $c(3, 1, 50)$. In the numeric version, the functions return the resulting matrix, the dominant eigenvalue (asymptotic population growth rate), and the corresponding stable stage distribution. In the literal version, the output is the projection matrix with all parameters retained in literal form, allowing for analytical inspection.
